# Supplementary material for: A curated dataset of great ape genome diversity
Source: Sci Data. 2025 Nov 19;12:1835. doi: 10.1038/s41597-025-06124-z (PMC12630759; doi:10.1038/s41597-025-06124-z)
Supplement: Supplementary file 1 — Supplementary Figures [file 41597_2025_6124_MOESM1_ESM.pdf]

Table of Contents: Legends for Supplementary Figures

Figure S1	Coverage distribution per individual, stratified by subspecies.	2
Figure S2	Coverage per chromosome for 22 autosomes, stratified by subspecies.	3
Figure S3	Coverage for sex chromosomes, stratified by subspecies and sex (chrY for males only, mitochondrial genome (chrM) for all individuals).	4
Figure S4	Last called position per chromosome, ensuring completeness of data, stratified by subspecies.	5
Figure S5	Number of non-reference genotype calls per chromosome, stratified by subspecies.	6
Figure S6	Number of heterozygous positions per chromosome, stratified by subspecies.	7
Figure S7	Transition-to-transversion ratio per chromosome, stratified by individual.	8
Figure S8	Principal Component Analysis for 4 PCs all Pan individuals, calculated on unfiltered sites.	9
Figure S9	Principal Component Analysis for 4 PCs all Pan troglodytes (chimpanzee) individuals, calculated on unfiltered sites.	10
Figure S10	Principal Component Analysis for 4 PCs all Pan paniscus (bonobo) individuals, calculated on unfiltered sites.	11
Figure S11	Principal Component Analysis for 4 PCs all Gorilla individuals, calculated on unfiltered sites.	12
Figure S12	Principal Component Analysis for 4 PCs all Pongo individuals, calculated on unfiltered sites.	13
Figure S13	ADMIXTURE clustering for all Pan individuals, for k=2 to k=10, calculated on 1,000,000 randomly chosen SNVs.	14
Figure S14	ADMIXTURE clustering for all Gorilla individuals, for k=1 to k=10, calculated on 1,000,000 randomly chosen SNVs.	15
Figure S15	ADMIXTURE clustering for all Pongo individuals, for k=1 to k=10, calculated on 1,000,000 randomly chosen SNVs.	16
Figure S16	Relatedness between all Pan individuals, as determined by KING in ngsRelate. Calculated on filtered dataset.	17
Figure S17	Relatedness between all Gorilla individuals, as determined by KING in ngsRelate. Calculated on filtered dataset.	18
Figure S18	Relatedness between all Pongo individuals, as determined by KING in ngsRelate. Calculated on filtered dataset.	19
Figure S19	Runs of Homozygosity (RoHs) in all individuals, as estimated by bcftools roh, stratified by subspecies and partitioned into short (50,000-250,000 bp), medium (250,000-1,000,000 bp) and long (>1,000,000) RoHs.	20
Figure S20	Subspecies assignment based on f3-statistics.	21
Figure S21	Geolocalization of chimpanzees based on rareCAGA.	22
Figure S22	Relatedness between all Gorilla individuals, including duplicated individuals that were removed from the dataset, as determined by KING in ngsRelate. Calculated on an unfiltered dataset.	23
Figure S23	Relatedness between all Pongo individuals, including duplicated individuals that were removed from the dataset, as determined by KING in ngsRelate. Calculated on an unfiltered dataset.	24
Figure S24	Relatedness between all Pan individuals, including duplicated individuals that were removed from the dataset, as determined by KING in ngsRelate. Calculated on an unfiltered dataset.	25

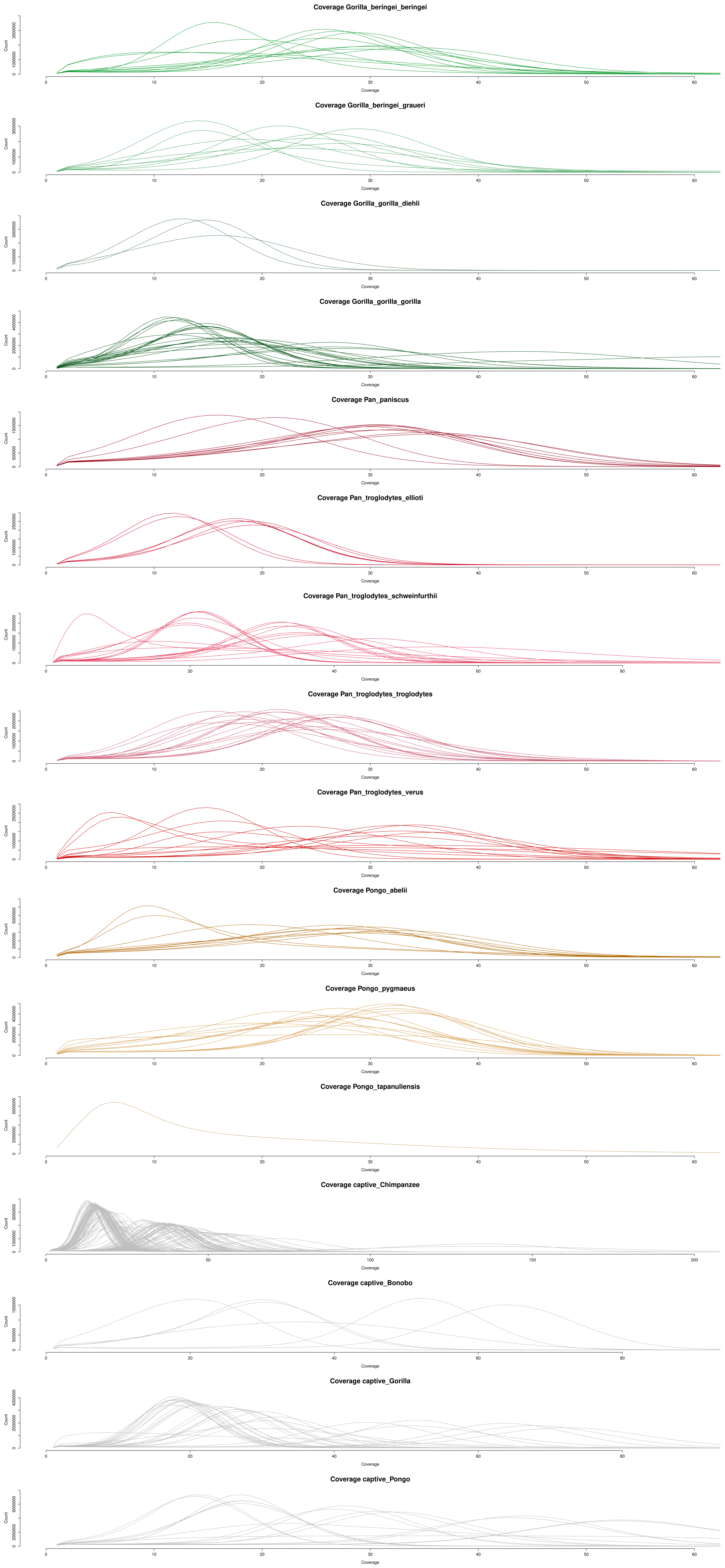

Figure S1. Coverage distribution per individual, stratified by subspecies.

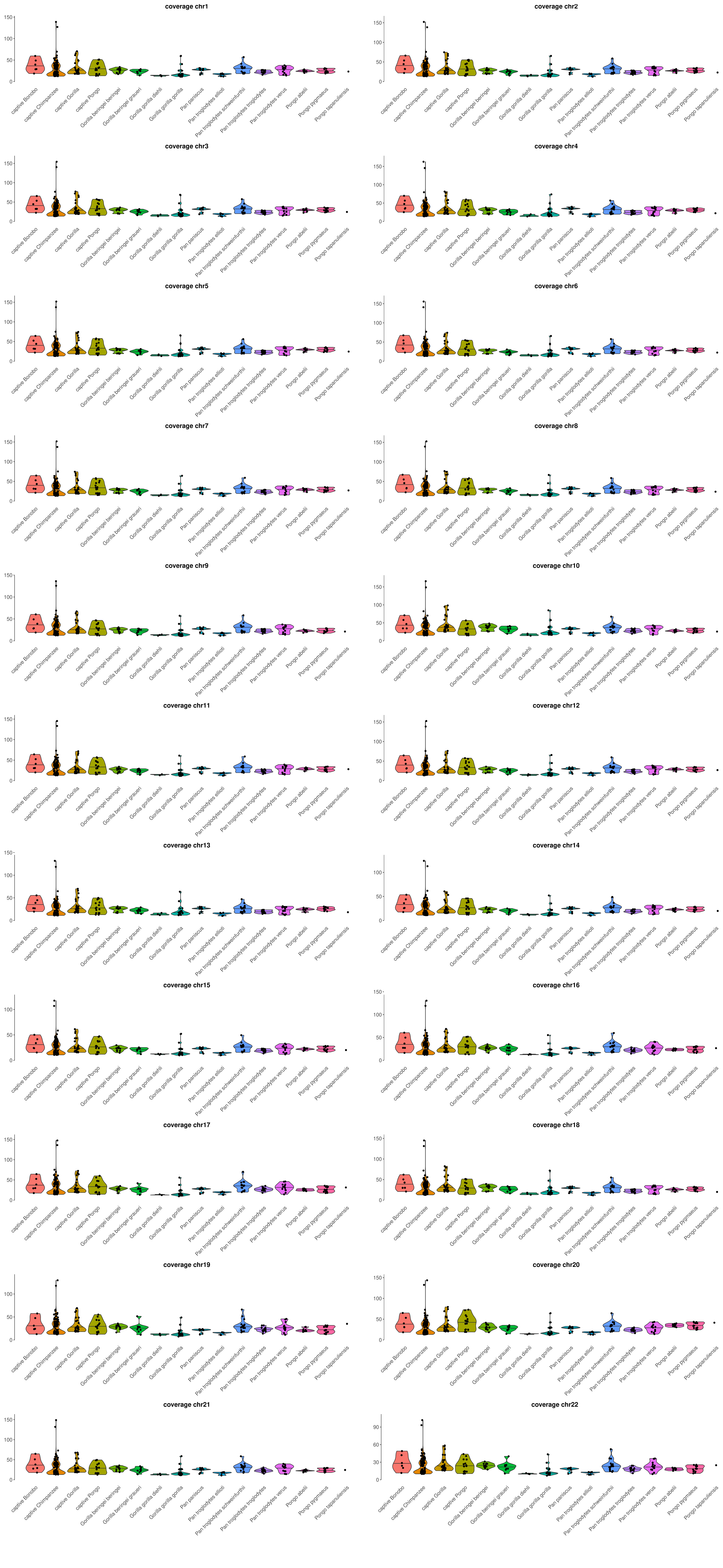

Figure S2. Coverage per chromosome for 22 autosomes, stratified by subspecies.

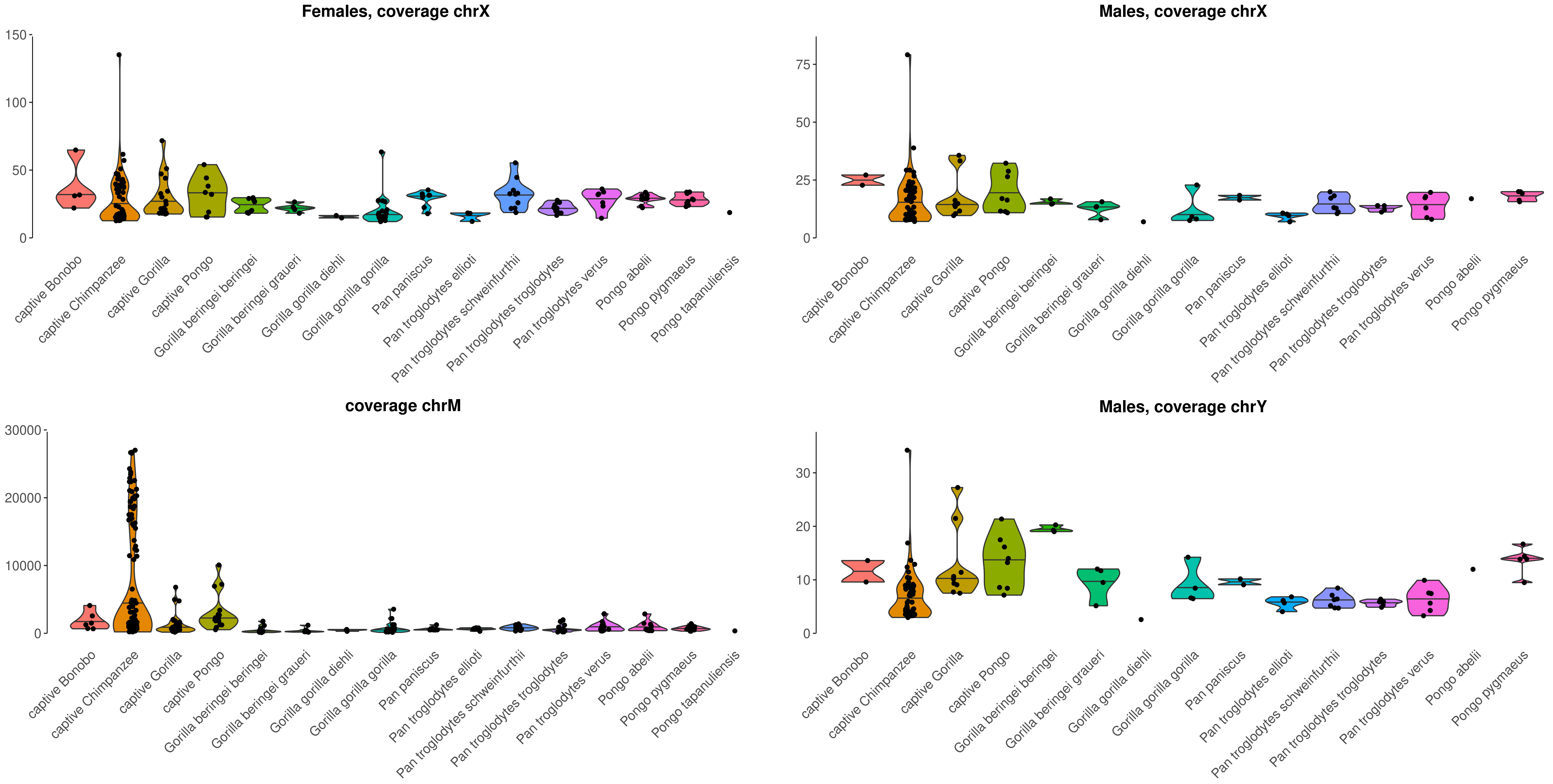

Figure S3. Coverage for sex chromosomes, stratified by subspecies and sex (chrY for males only, mitochondrial genome (chrM) for all individuals).

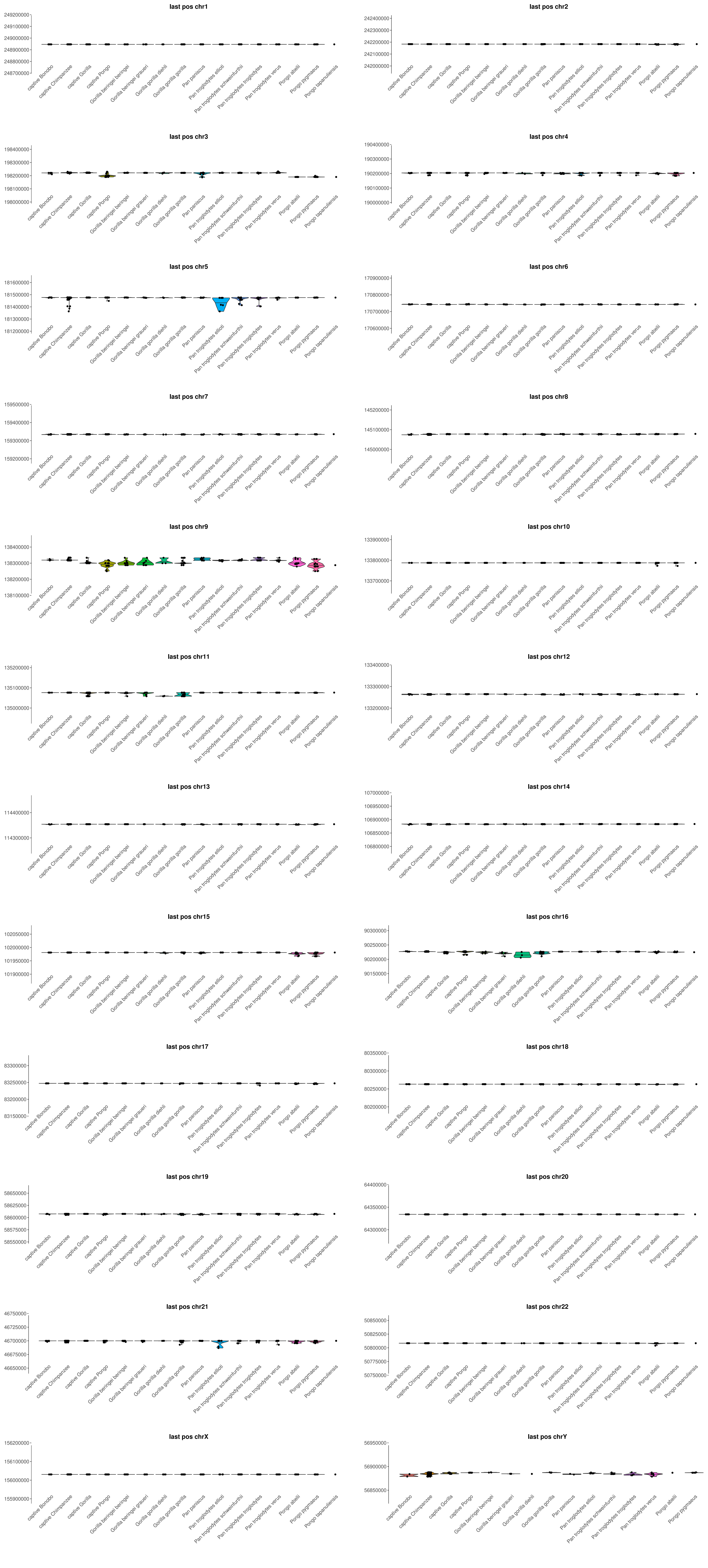

Figure S4. Last called position per chromosome, ensuring completeness of data, stratified by subspecies.

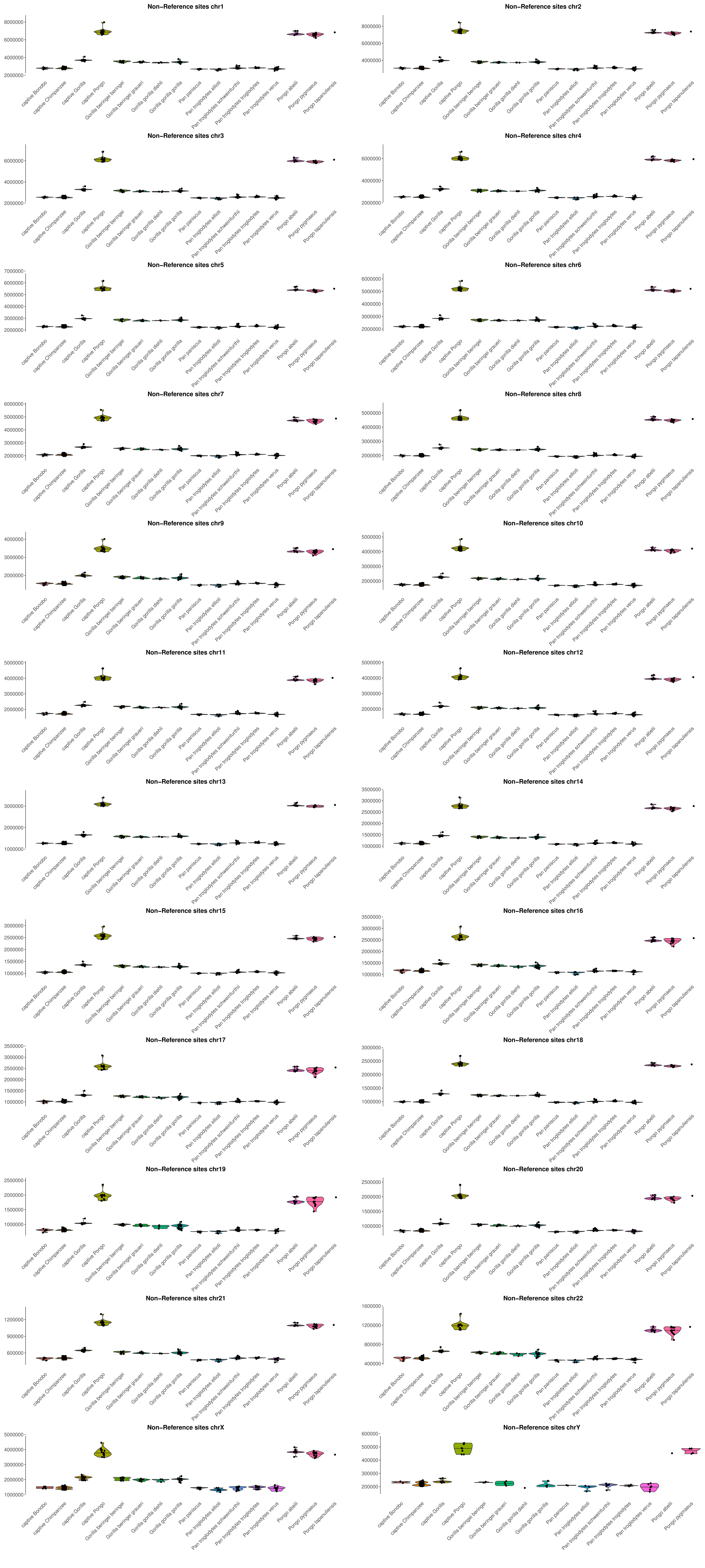

Figure S5. Number of non-reference genotype calls per chromosome, stratified by subspecies.

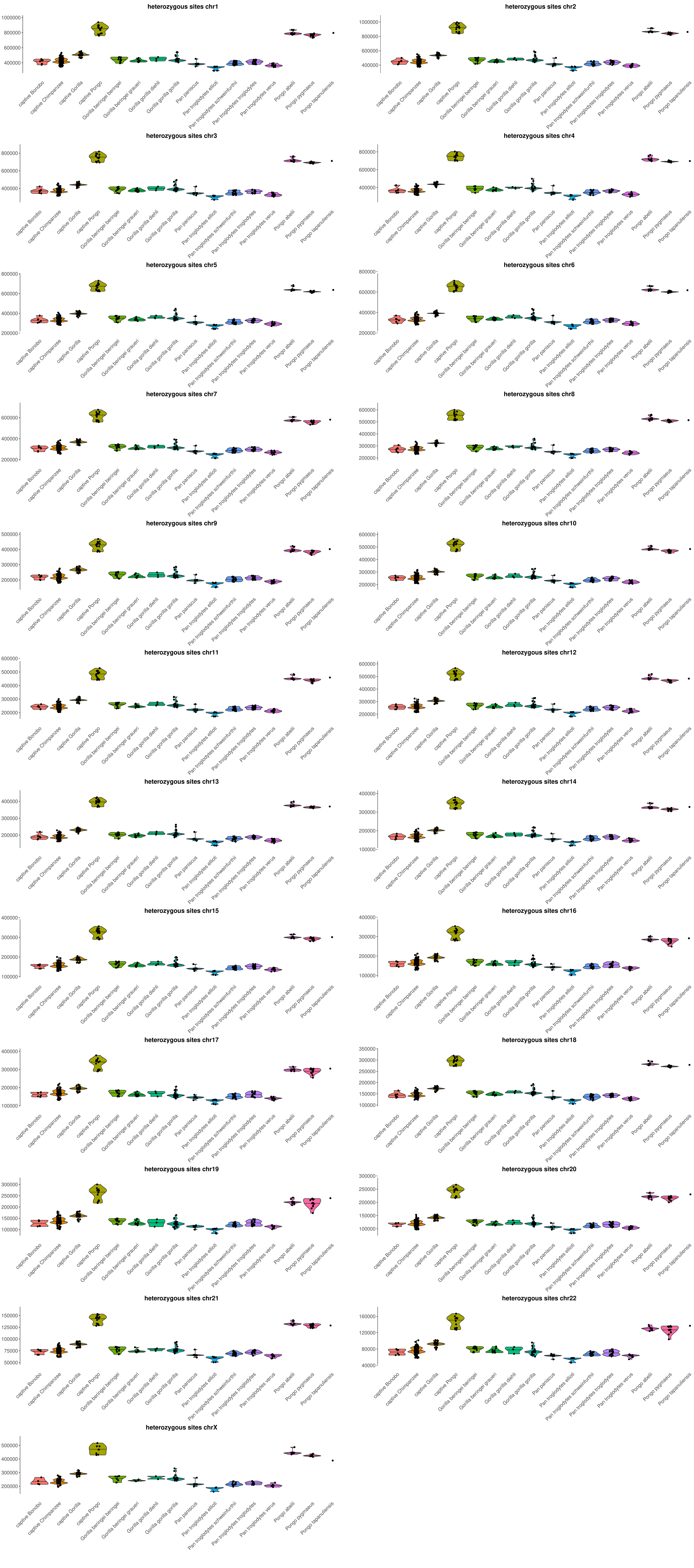

Figure S6. Number of heterozygous positions per chromosome, stratified by subspecies.

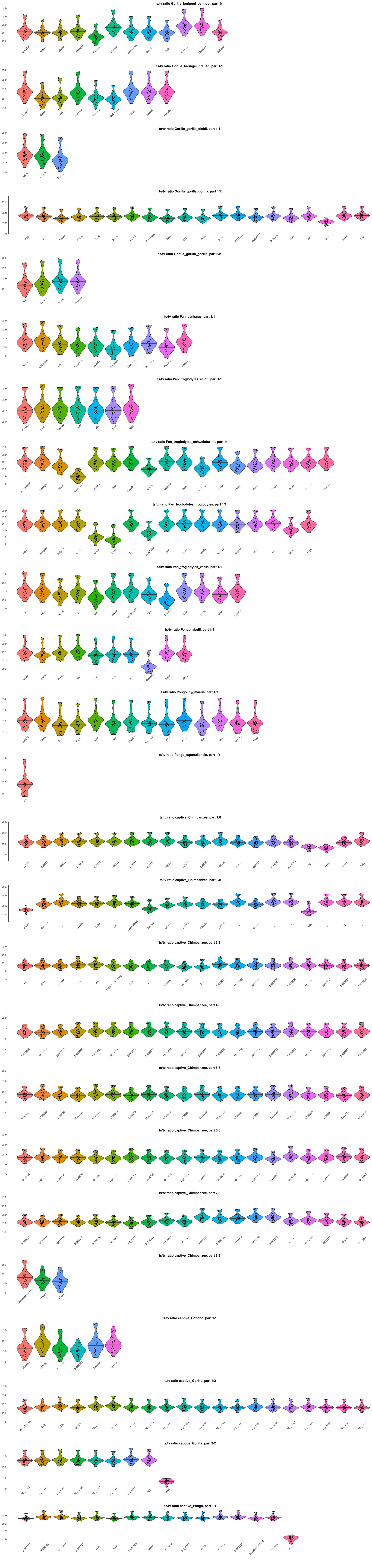

Figure S7. Transition-to-transversion ratio per chromosome, stratified by individual.

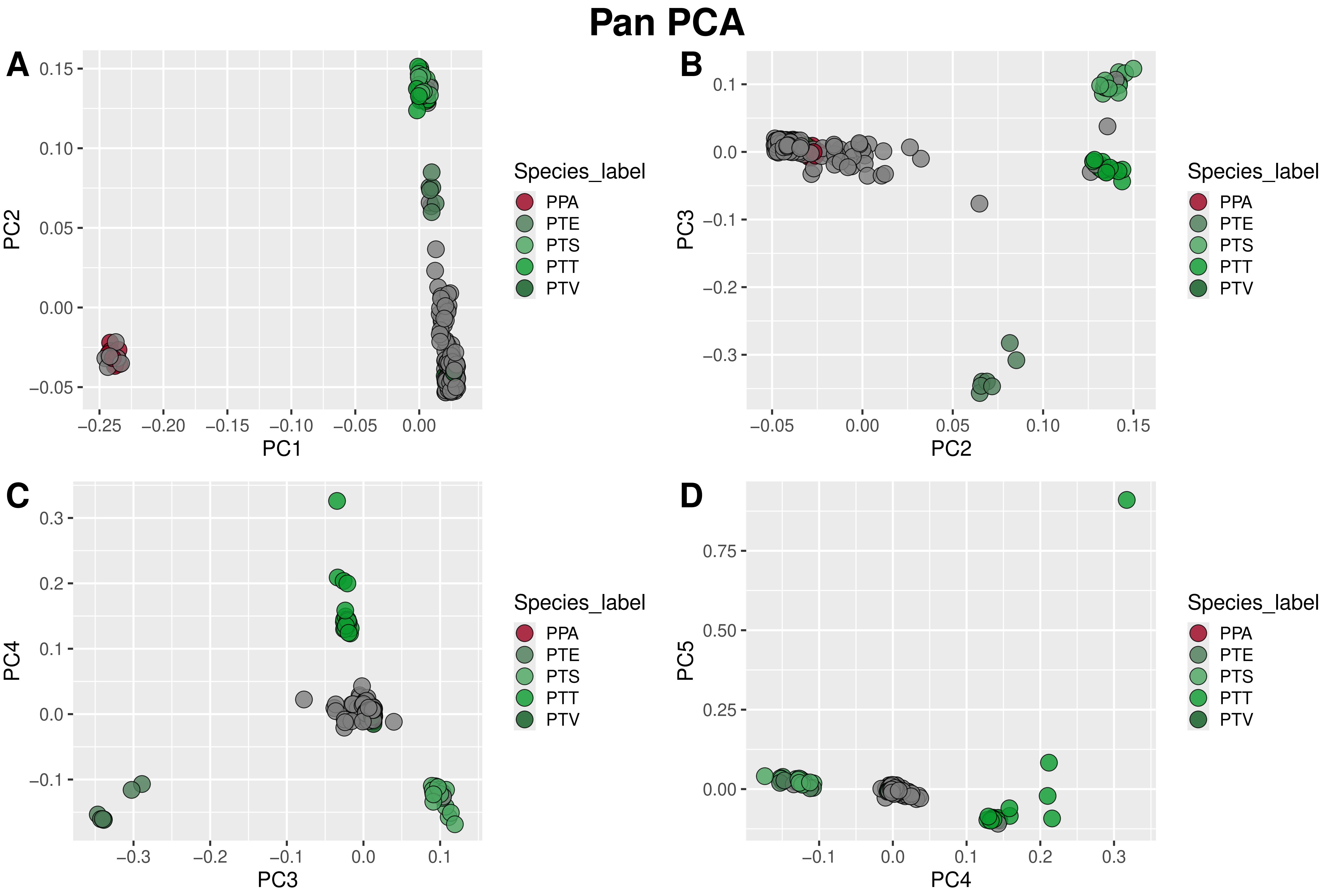

Figure S8. Principal Component Analysis for 4 PCs all Pan individuals, calculated on unfiltered sites.

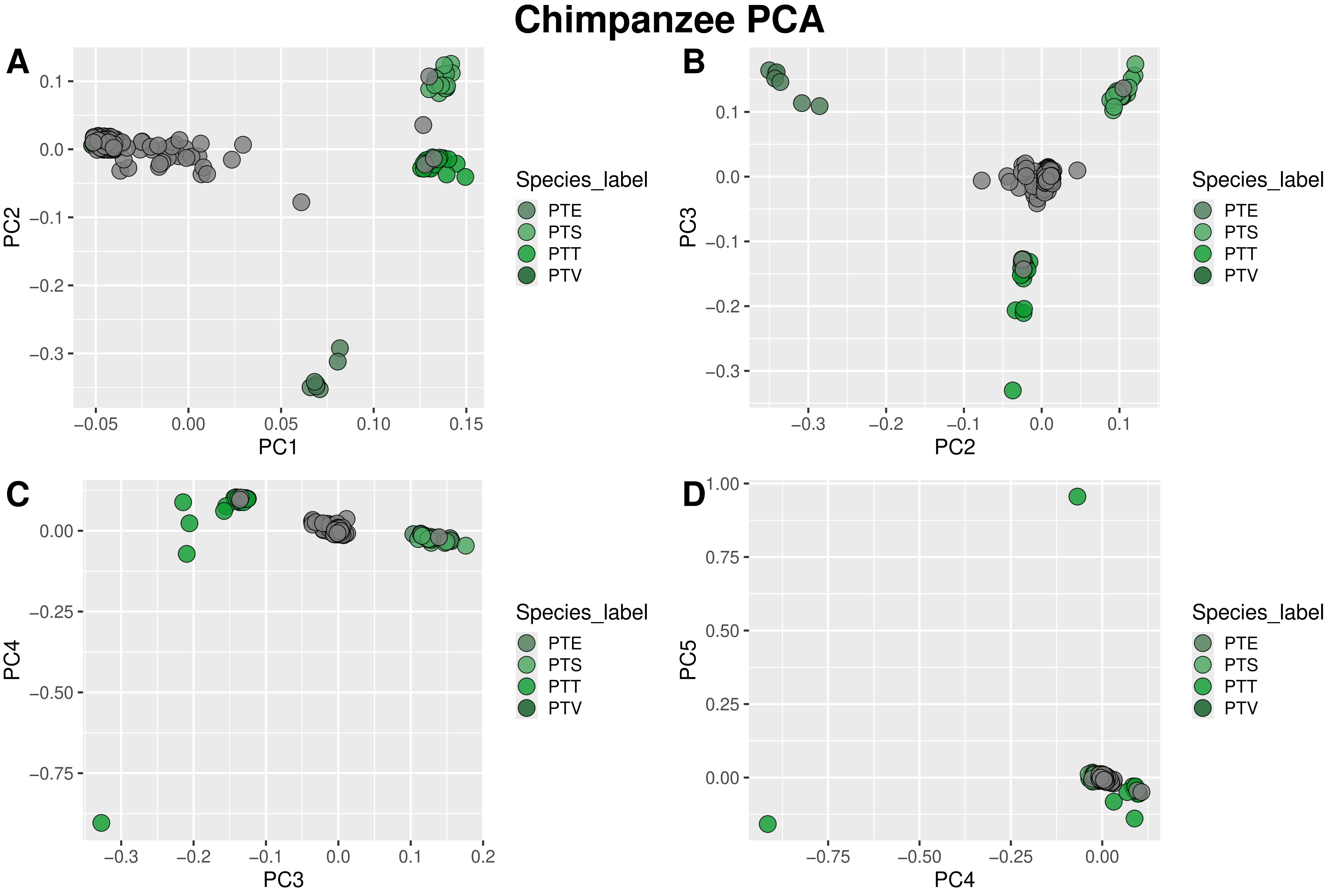

Figure S9. Principal Component Analysis for 4 PCs all Pan troglodytes (chimpanzee) individuals, calculated on unfiltered sites.

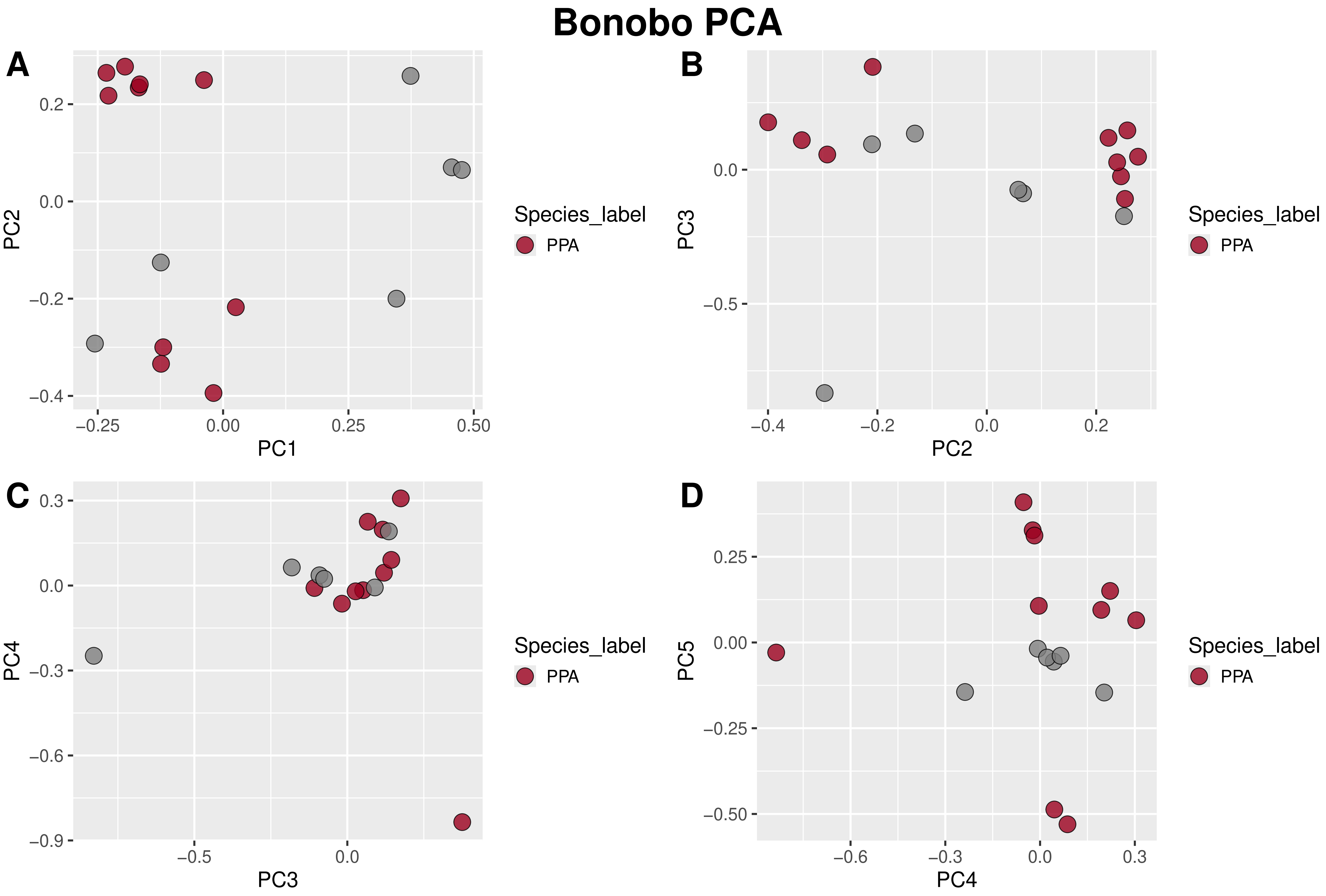

Figure S10. Principal Component Analysis for 4 PCs all Pan paniscus (bonobo) individuals, calculated on unfiltered sites.

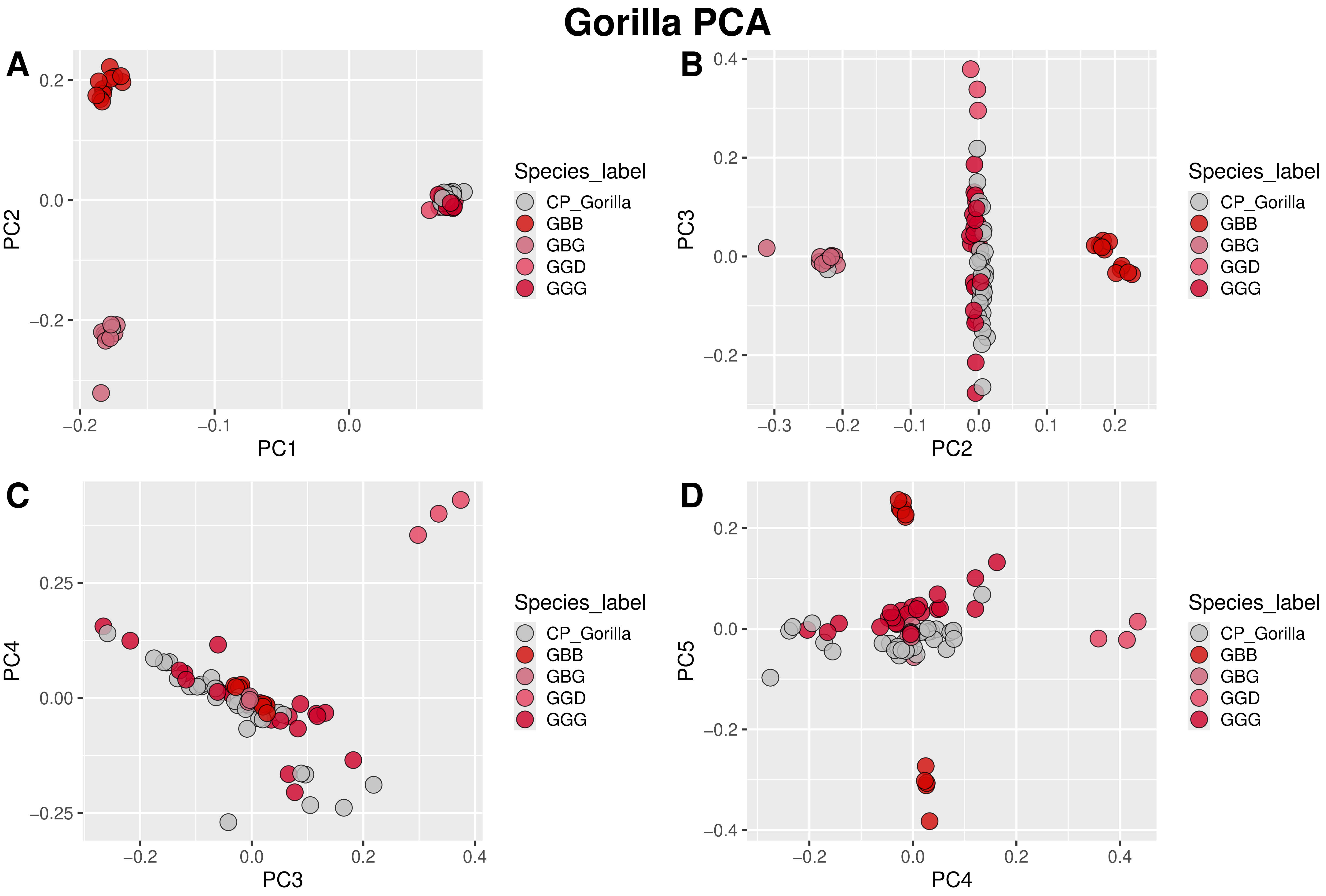

Figure S11. Principal Component Analysis for 4 PCs all Gorilla individuals, calculated on unfiltered sites.

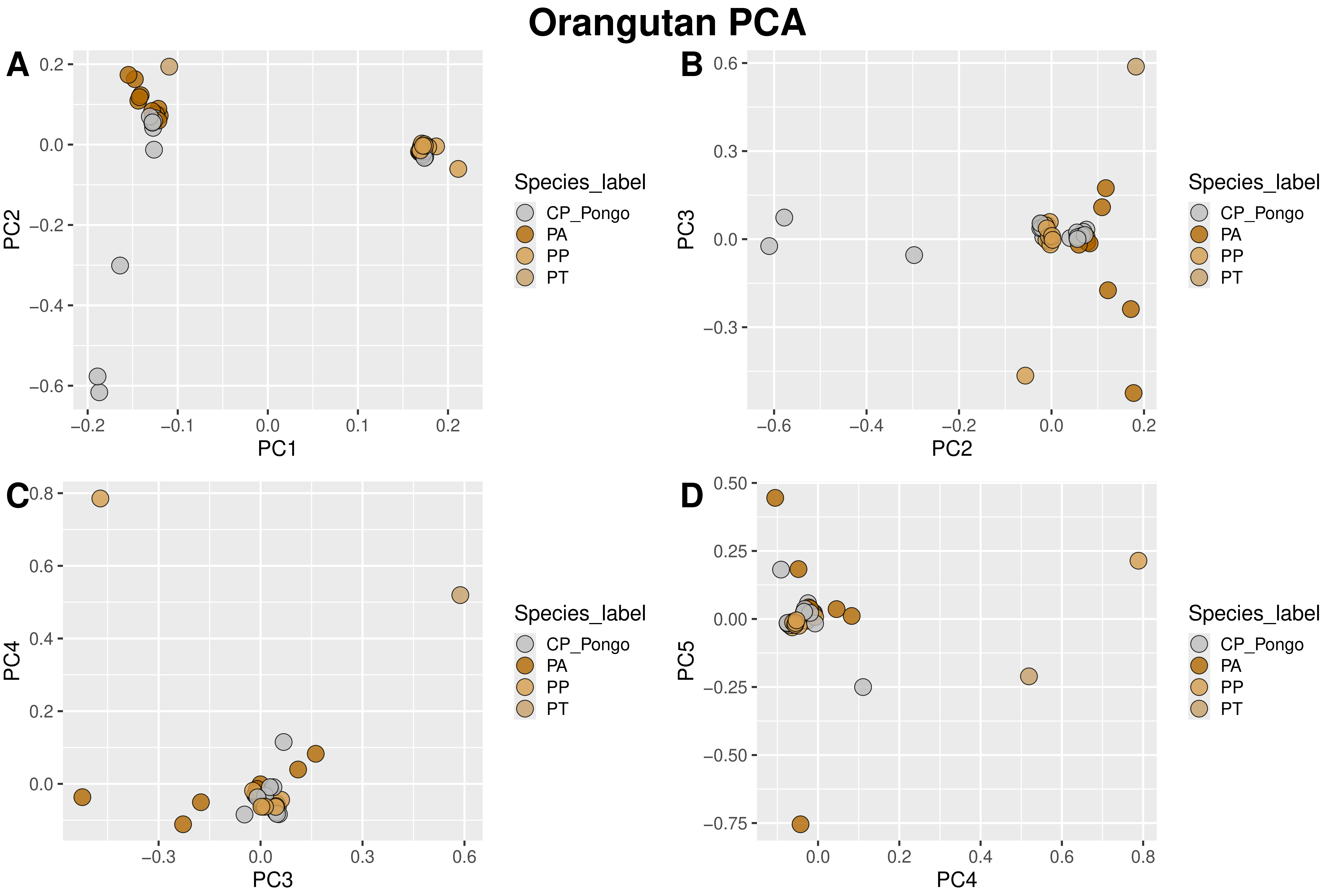

Figure S12. Principal Component Analysis for 4 PCs all Pongo individuals, calculated on unfiltered sites.

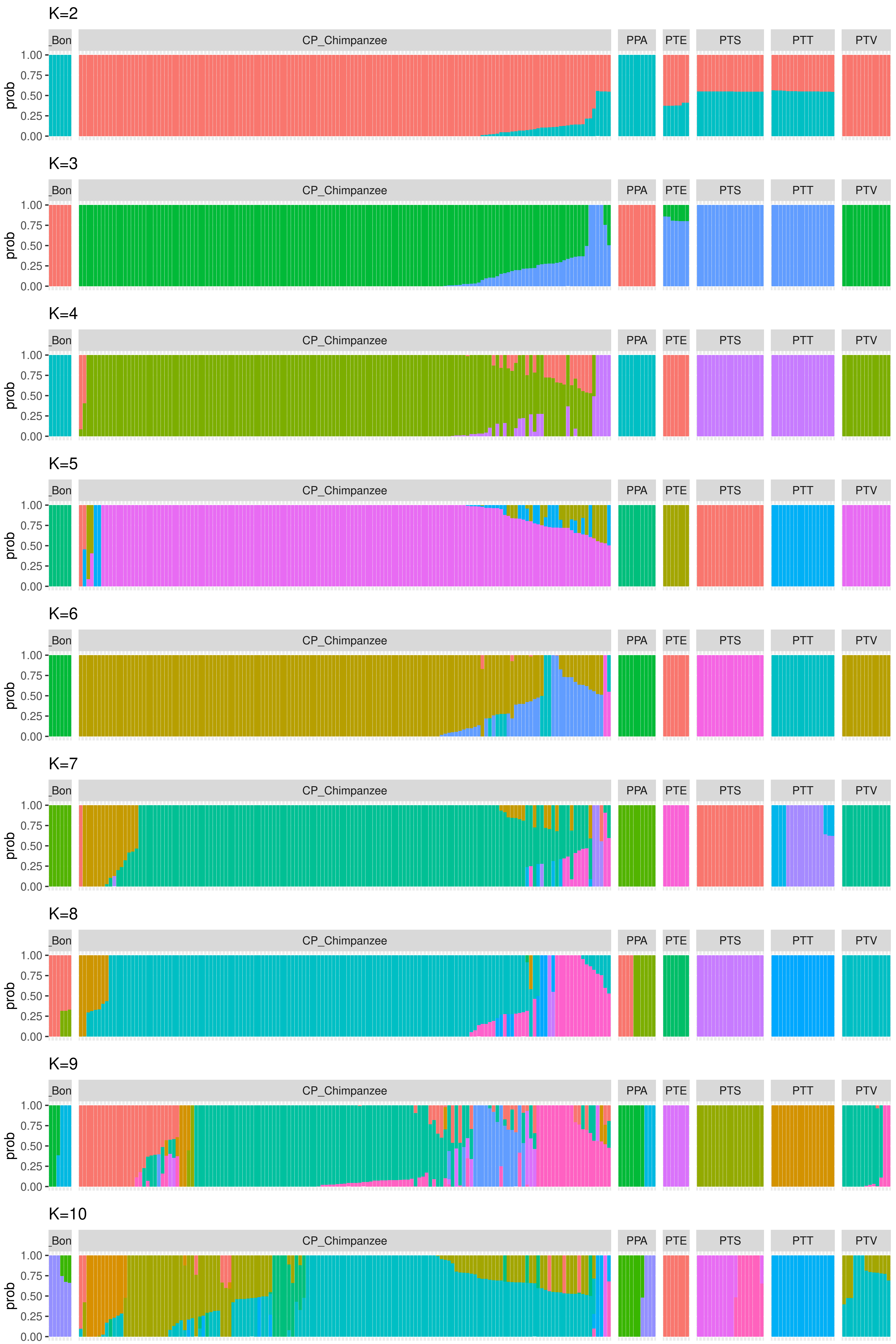

Figure S13. ADMIXTURE clustering for all Pan individuals, for k=2 to k=10, calculated on 1,000,000 randomly chosen SNVs.

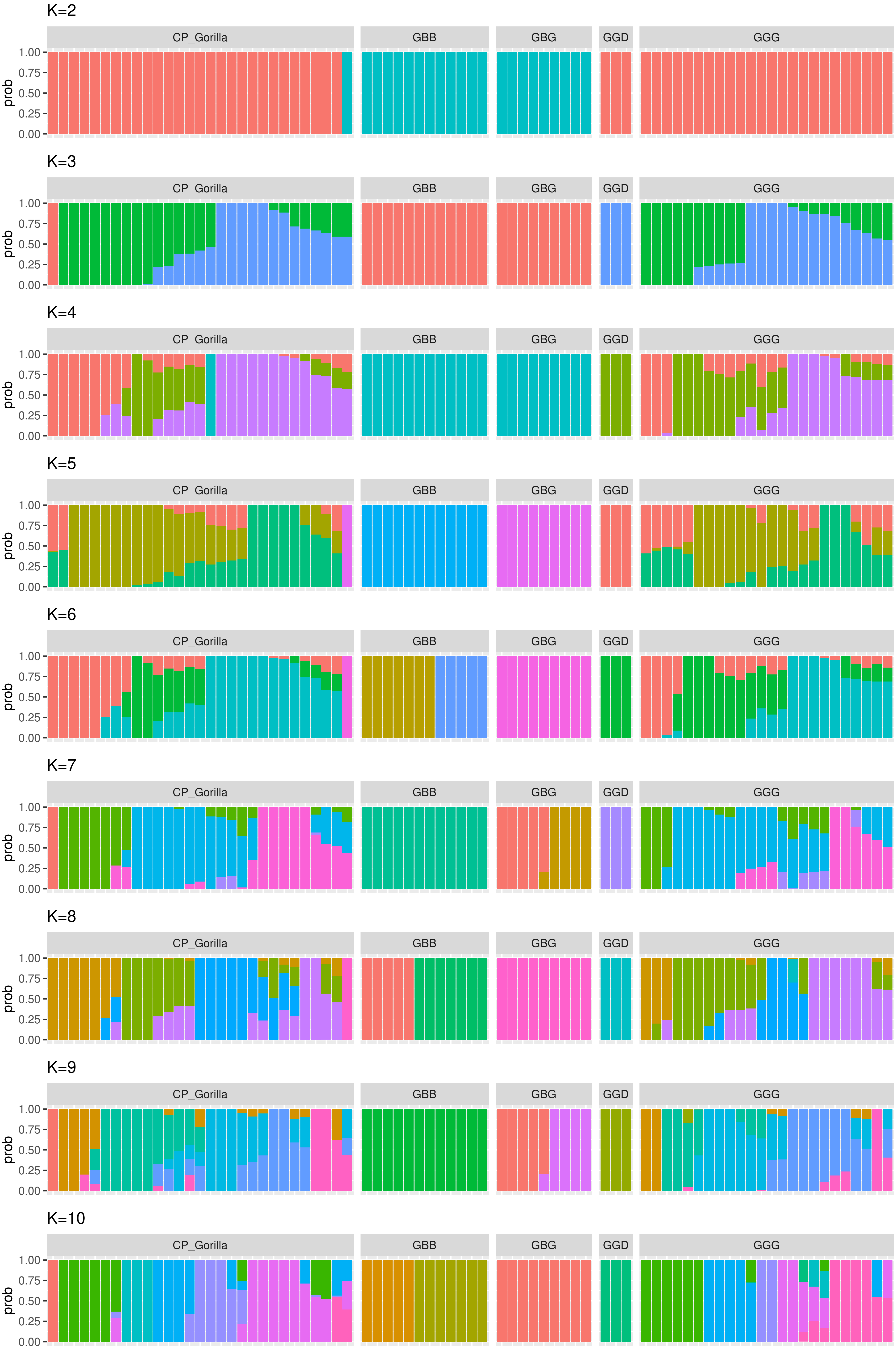

Figure S14. ADMIXTURE clustering for all Gorilla individuals, for k=1 to k=10, calculated on 1,000,000 randomly chosen SNVs.

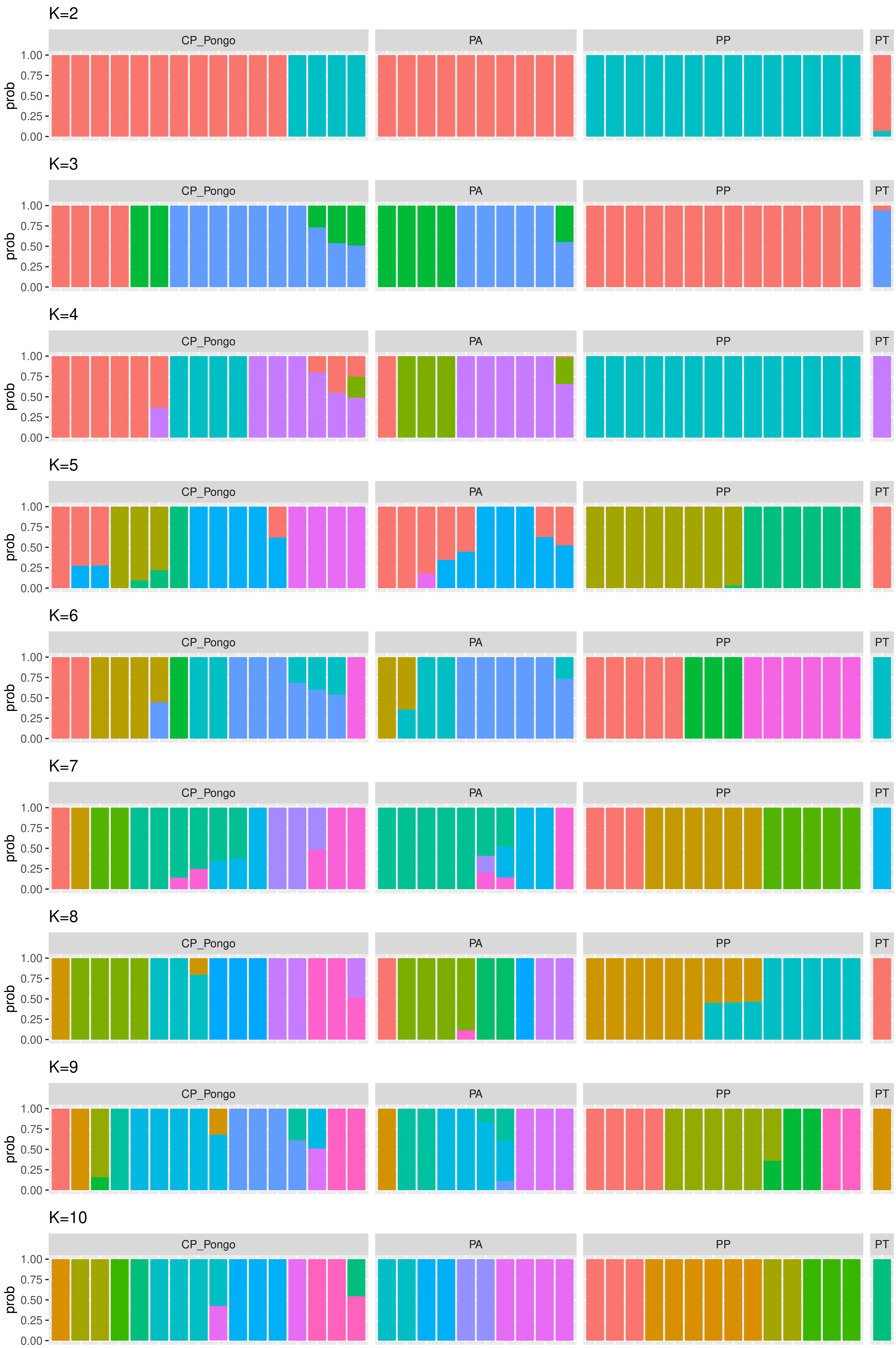

Figure S15. ADMIXTURE clustering for all Pongo individuals, for k=1 to k=10, calculated on 1,000,000 randomly chosen SNVs.

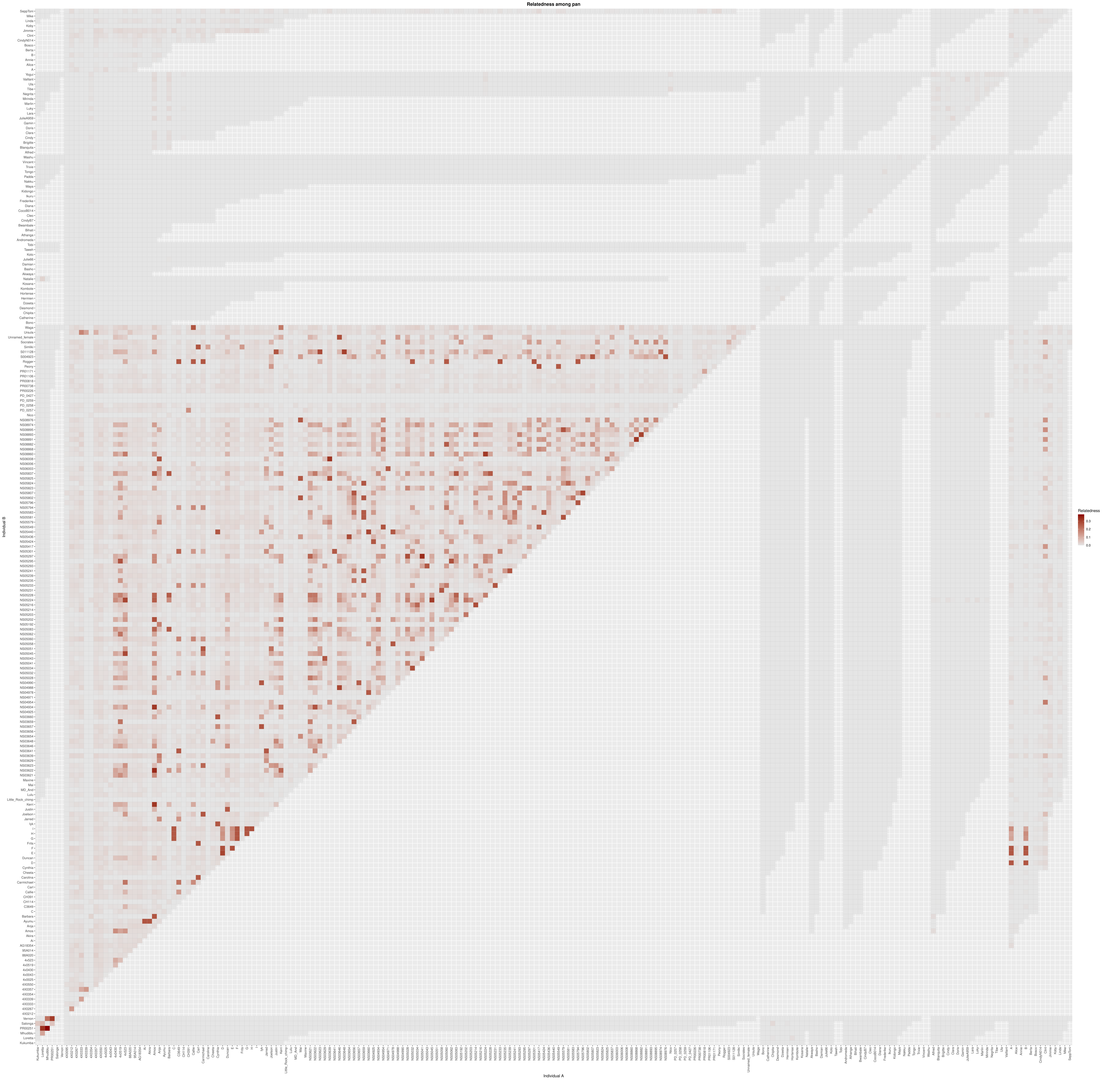

Figure S16. Relatedness between all Pan individuals, as determined by KING in ngsRelate. Calculated on filtered dataset.

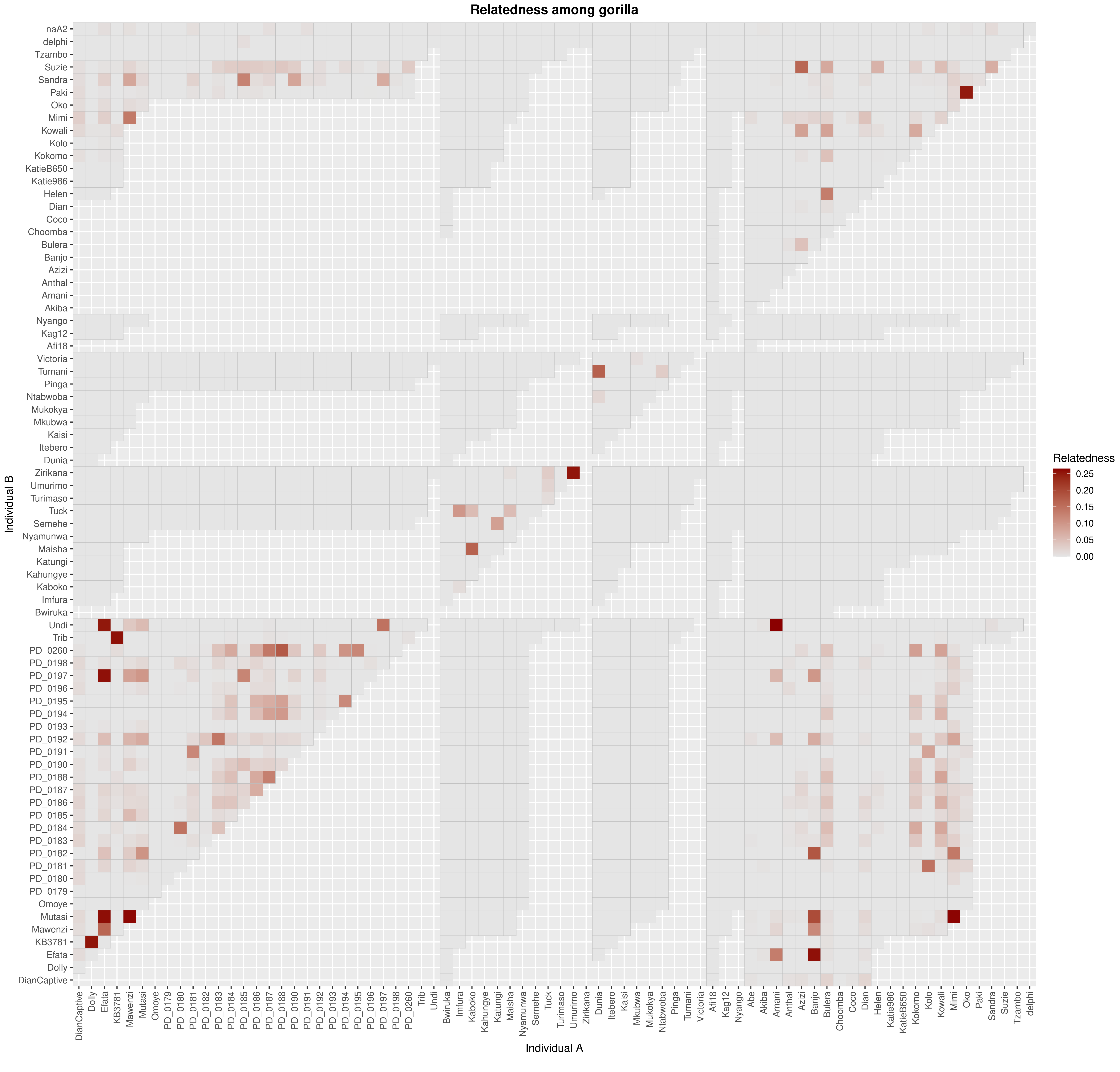

Figure S17. Relatedness between all Gorilla individuals, as determined by KING in ngsRelate. Calculated on filtered dataset.

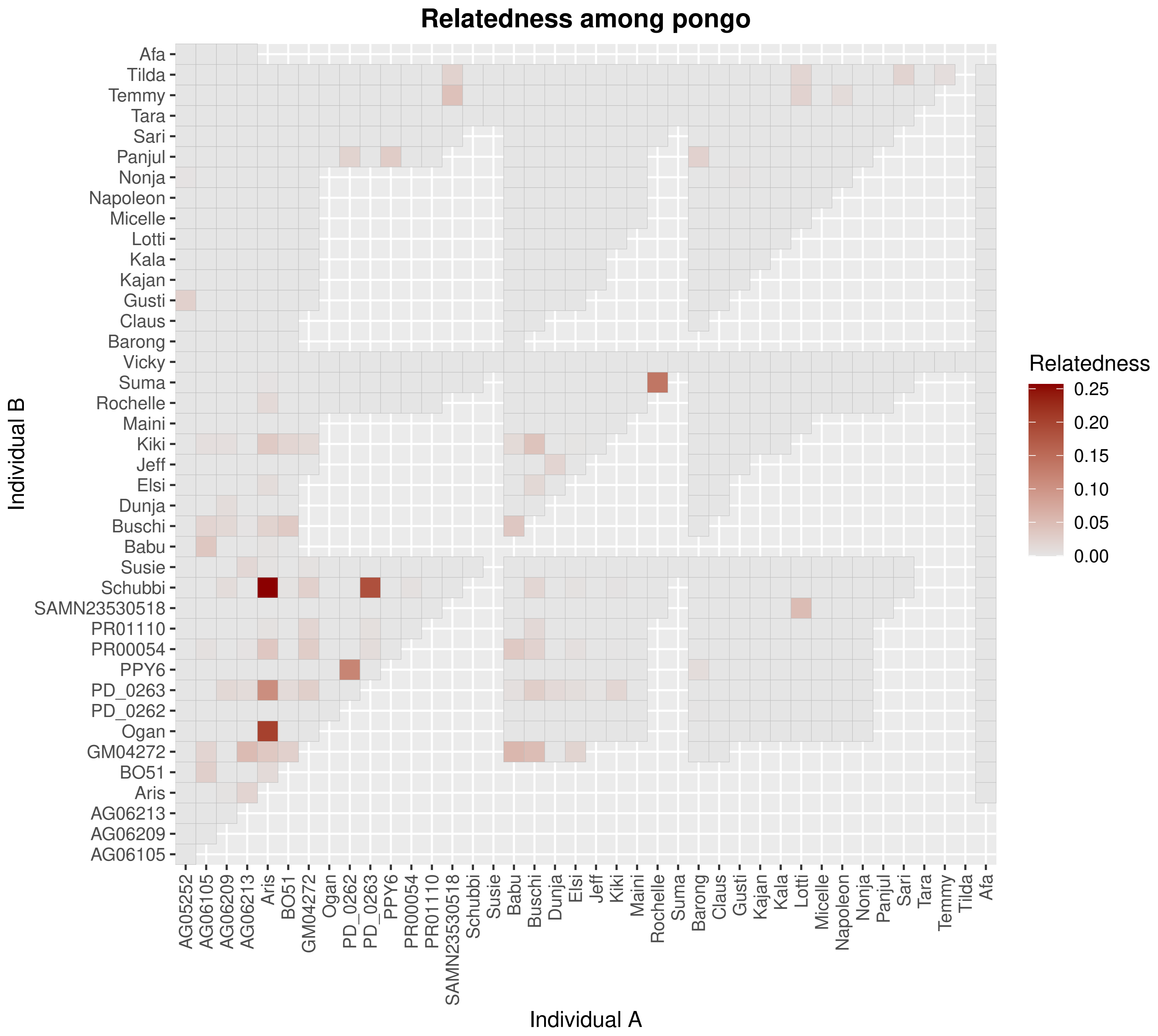

Figure S18. Relatedness between all Pongo individuals, as determined by KING in ngsRelate. Calculated on filtered dataset.

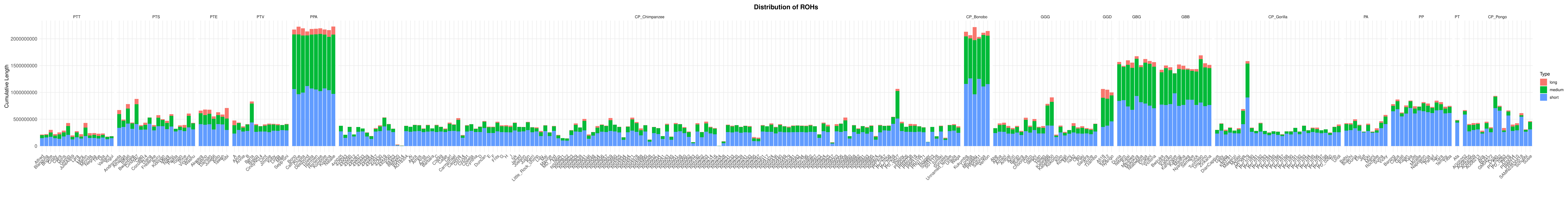

Figure S19. Runs of Homozygosity (RoHs) in all individuals, as estimated by bcftools roh, stratified by subspecies and partitioned into short (50,000-250,000 bp), medium (250,000-1,000,000 bp) and long (>1,000,000) RoHs.

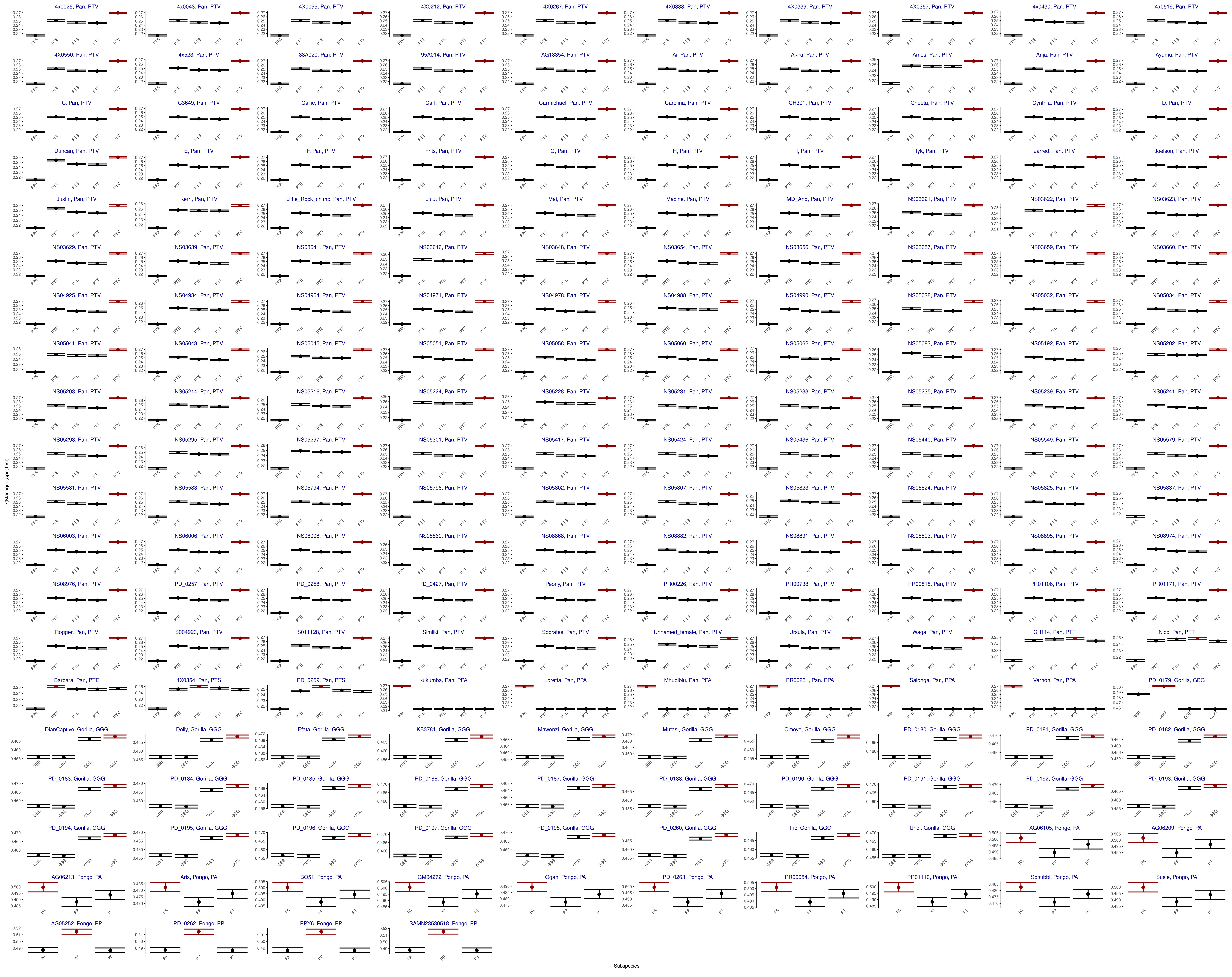

Figure S20. Subspecies assignment based on f3-statistics.

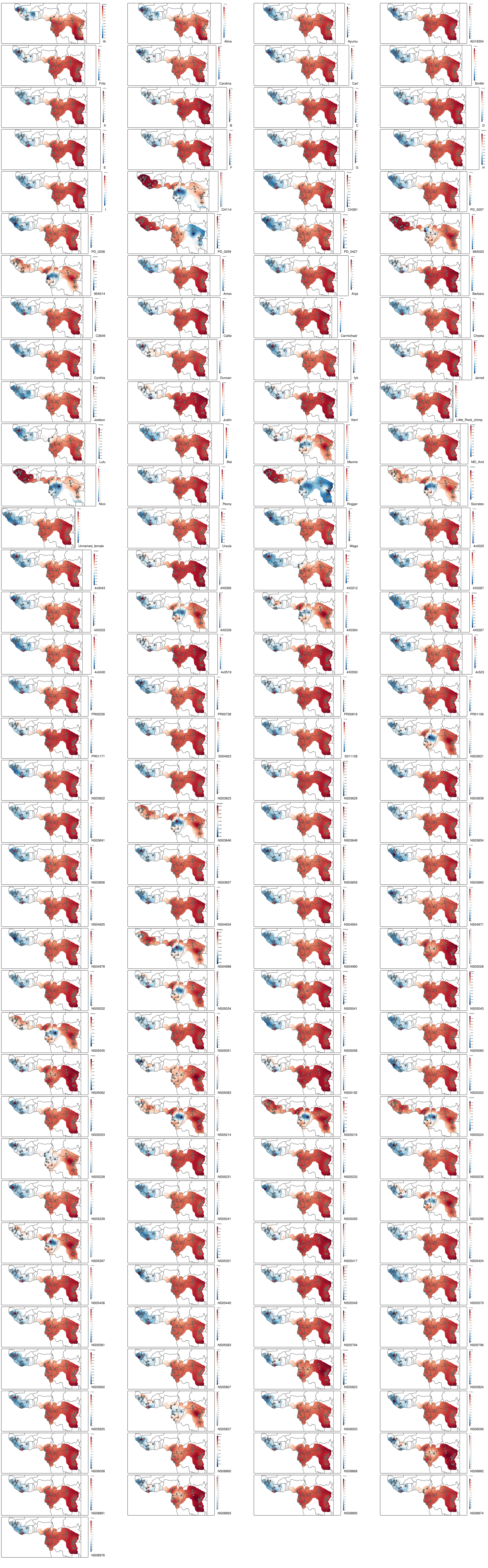

Figure S21. Geolocalization of chimpanzees based on rareCAGA.

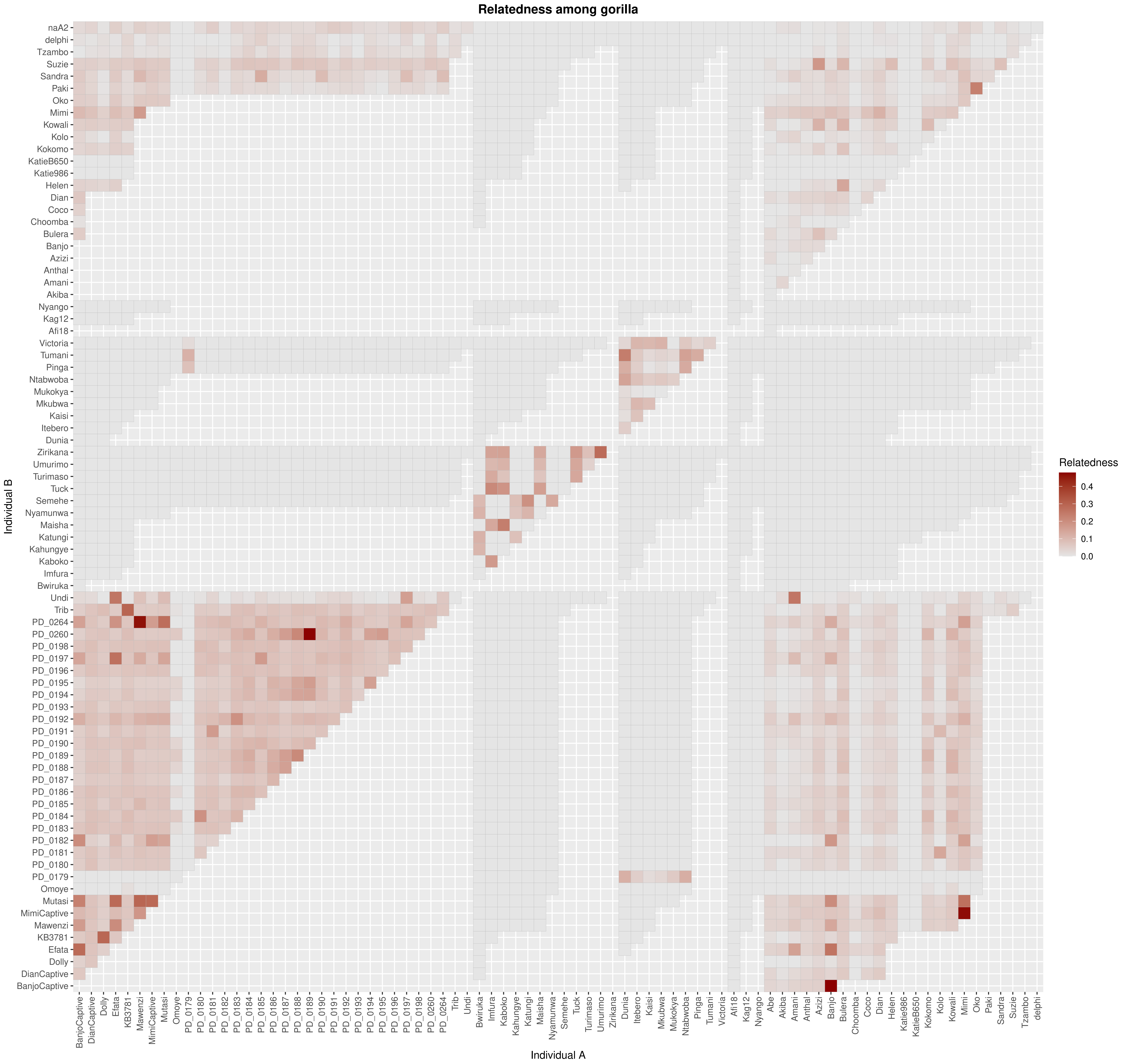

Figure S22. Relatedness between all Gorilla individuals, including duplicated individuals that were removed from the dataset, as determined by KING in ngsRelate. Calculated on an unfiltered dataset.

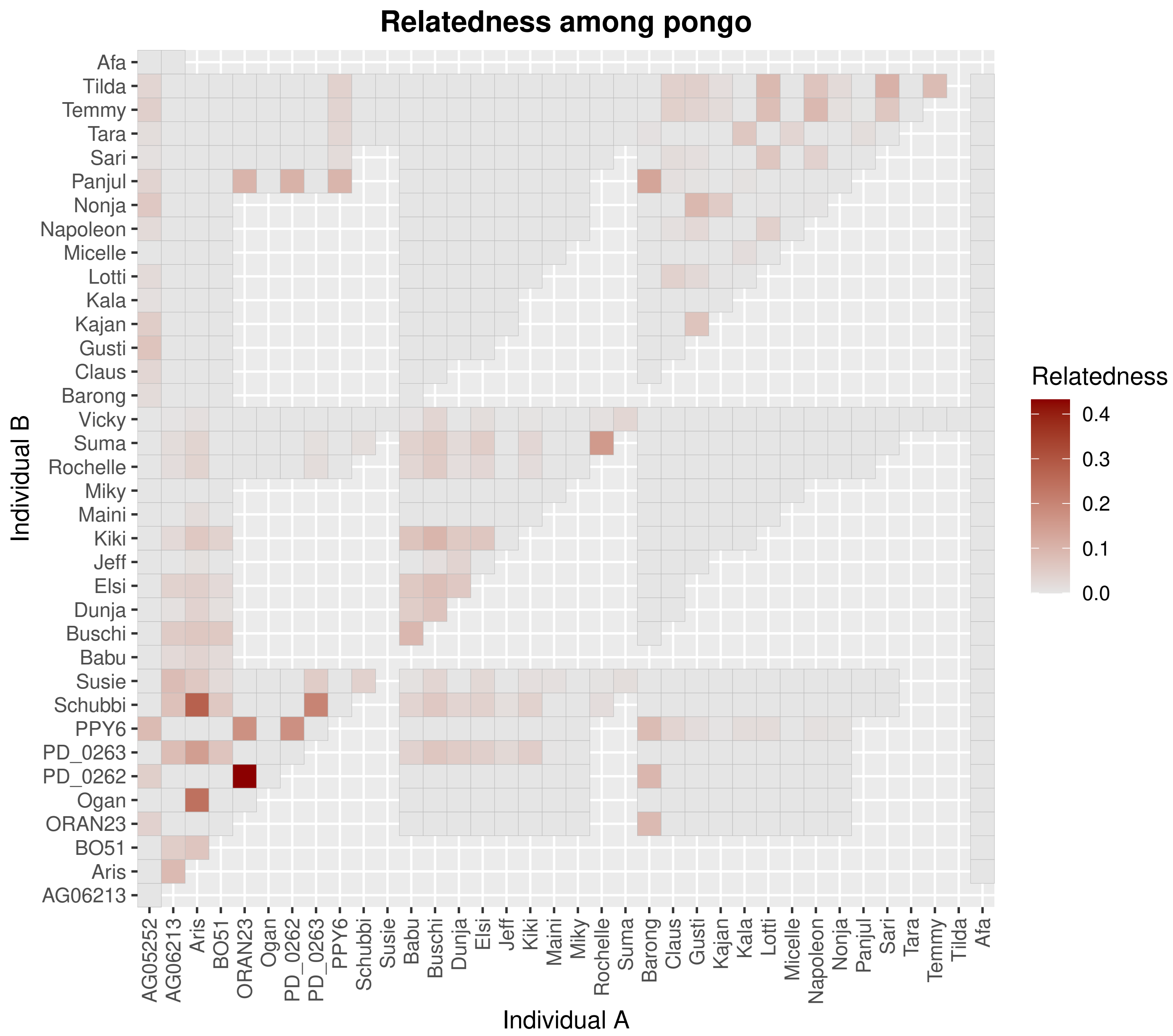

Figure S23. Relatedness between all Pongo individuals, including duplicated individuals that were removed from the dataset, as determined by KING. Calculated on an unfiltered dataset.

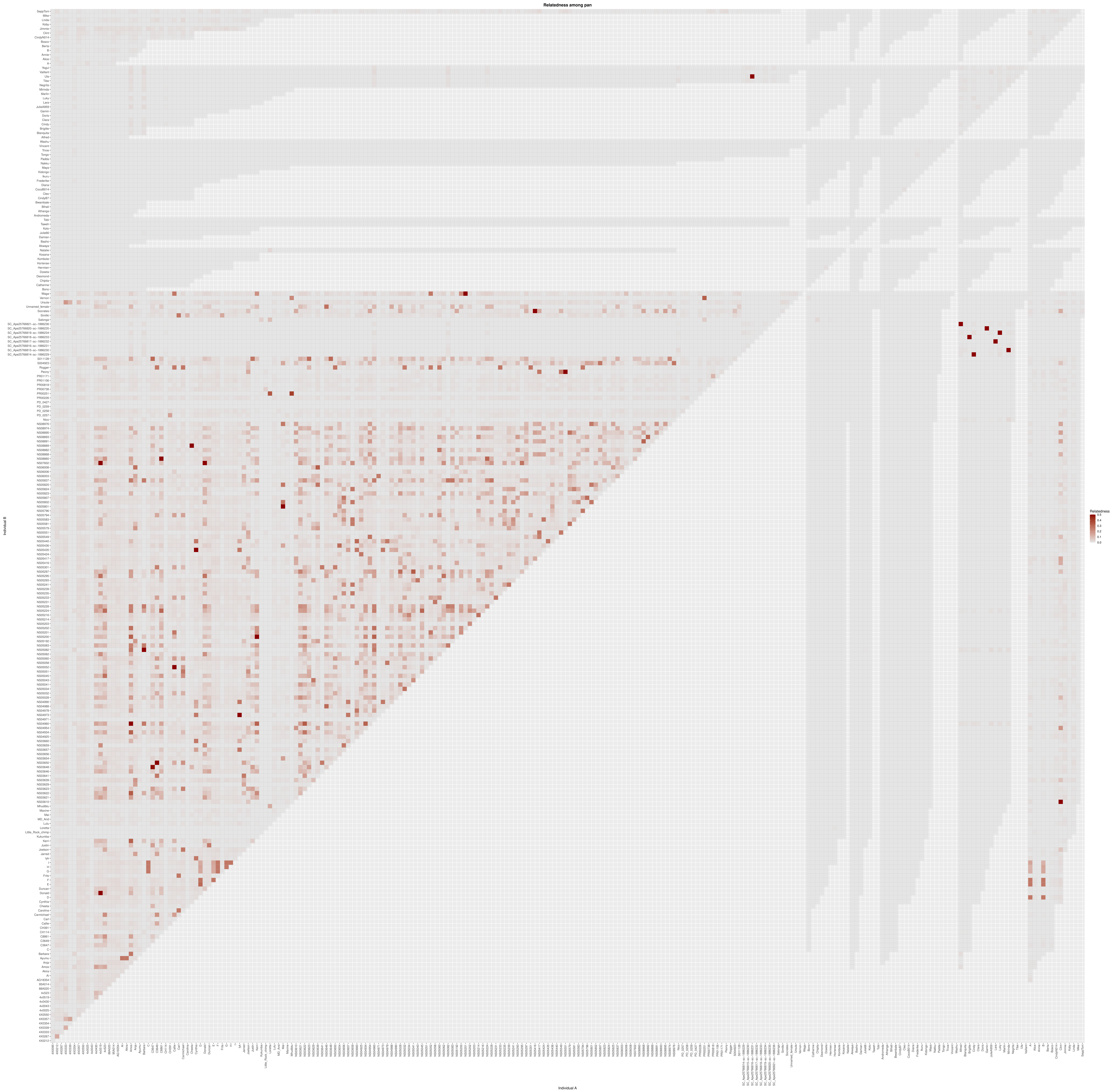

Figure S24. Relatedness between all Pan individuals, including duplicated individuals that were removed from the dataset, as determined by KING in ngsRelate. Calculated on an unfiltered dataset.
